# Supplementary material for: Neodymium isotope evidence for glacial-interglacial variability of deepwater transit time in the Pacific Ocean
Source: Nat Commun. 2018 Nov 9;9:4709. doi: 10.1038/s41467-018-07079-z (PMC6226442; doi:10.1038/s41467-018-07079-z)
Supplement: Supplementary file 2 — Supplementary Information [file 41467_2018_7079_MOESM2_ESM.pdf]

Neodymium isotope evidence for glacial-interglacial variability of deepwater transit time in the Pacific Ocean

Hu et al.

Contents:

Supplementary Figure 1: Sedimentary records of V21-30.

Supplementary Figure 2: Sedimentary records of ODP1241.

Supplementary Figure 3: Modelled porewater ε_{Nd} records of V21-30 and ODP1241 in the sensitivity tests compared to foraminiferal ε_{Nd} records.

Supplementary Figure 4: Sensitivity tests of deep Pacific transit time.

Supplementary Table 1: Locations of the cores used in this study.

Supplementary Table 2: Nd isotope record of V21-30.

Supplementary Table 3: Nd isotope record of V28-239.

Supplementary Table 4: Nd isotope record of SO136-38.

Supplementary Table 5: Nd isotope record of CHAT16K.

Supplementary Table 6: Nd isotope record of ODP1241.

Supplementary Table 7: Nd isotope record of ODP846.

Supplementary Table 8: Nd isotope record of RC13-114.

Supplementary Table 9: Radiocarbon ages of the upper part of ODP1241.

Supplementary Table 10: List of parameters, corresponding abbreviations, and their values used in this study.

Supplementary Figures

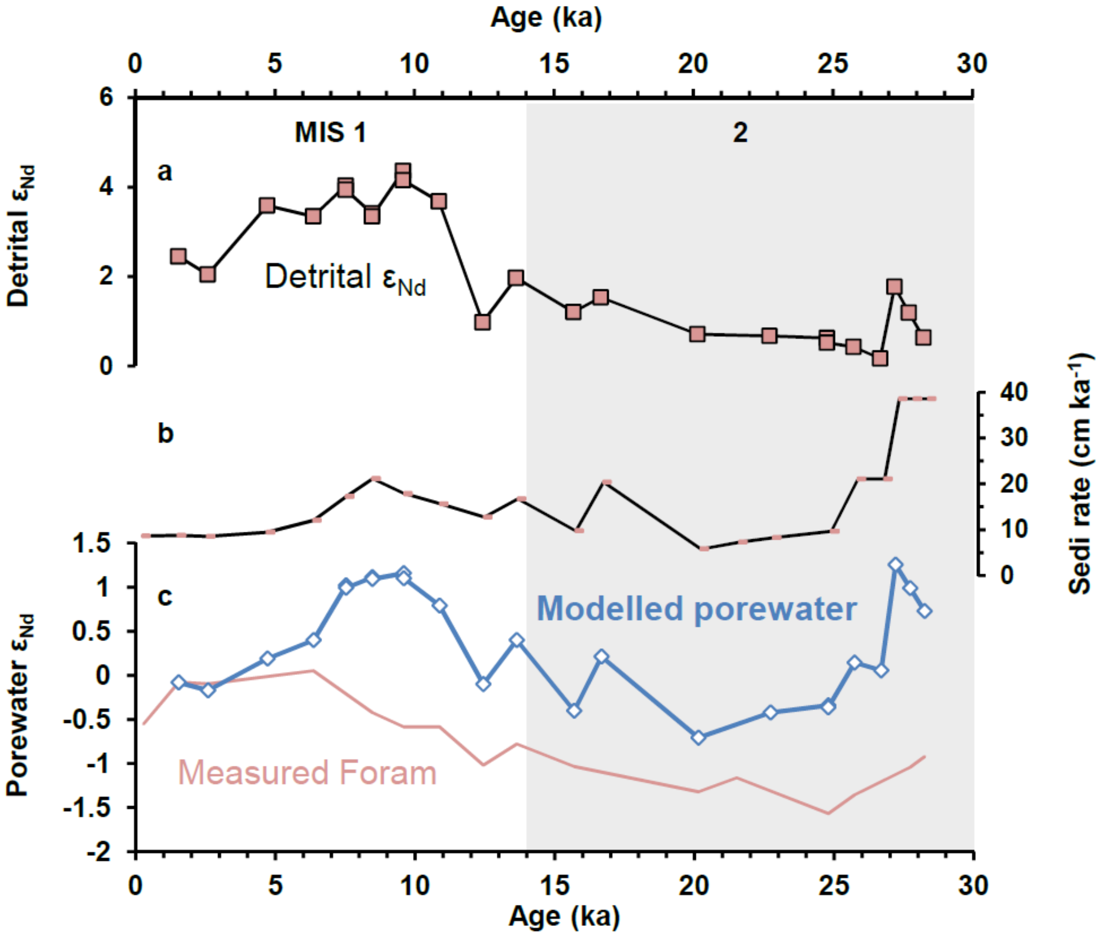

Supplementary Figure 1: Sedimentary records of V21-30. (a) Detrital ε_{Nd} record. (b) Sedimentation rate record. (c) Modelled porewater ε_{Nd} record (blue) in comparison with measured foraminiferal ε_{Nd} (pink). Glacial MIS 2 is highlighted in grey.

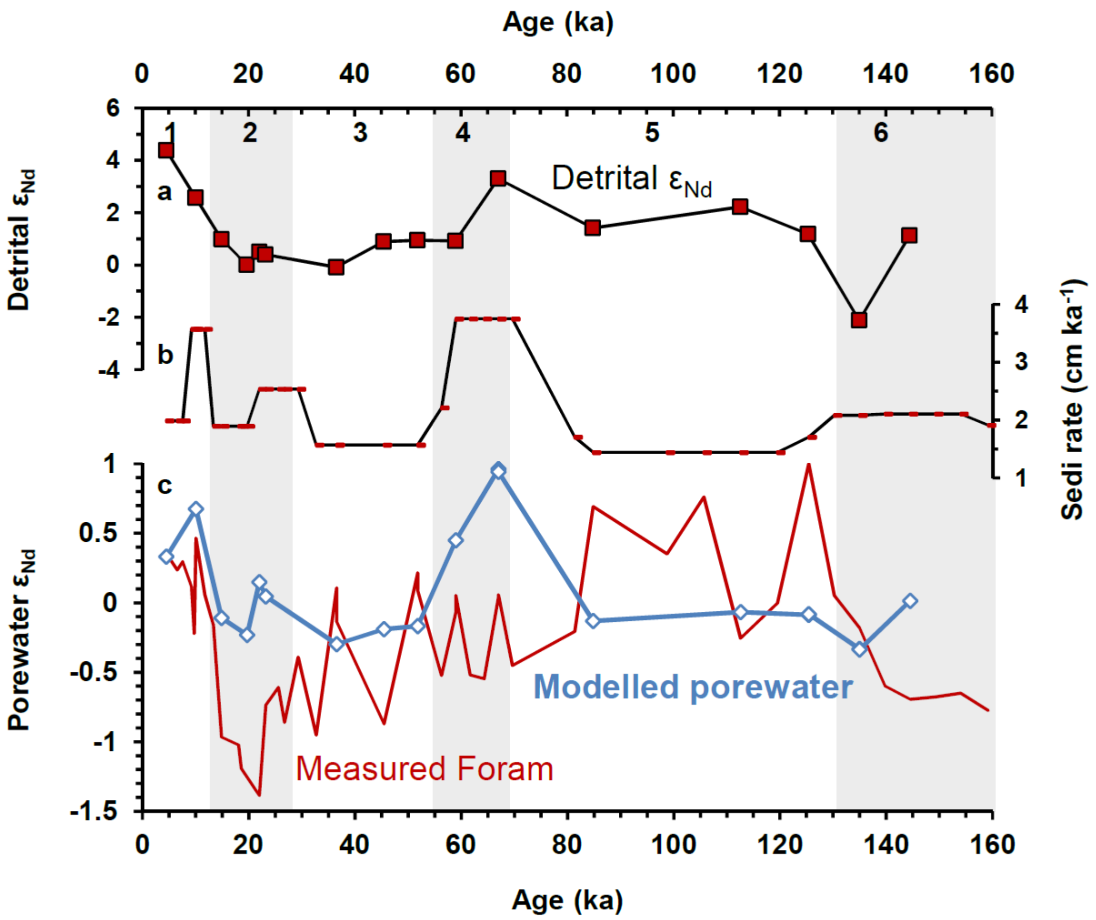

Supplementary Figure 2: Sedimentary records of ODP1241. (a) Detrital ε_{Nd} record. (b) Sedimentation rate record. (c) Modelled porewater ε_{Nd} record (blue) in comparison with measured foraminiferal ε_{Nd} (red). Glacial MIS 2, 4 and 6 are highlighted in grey.

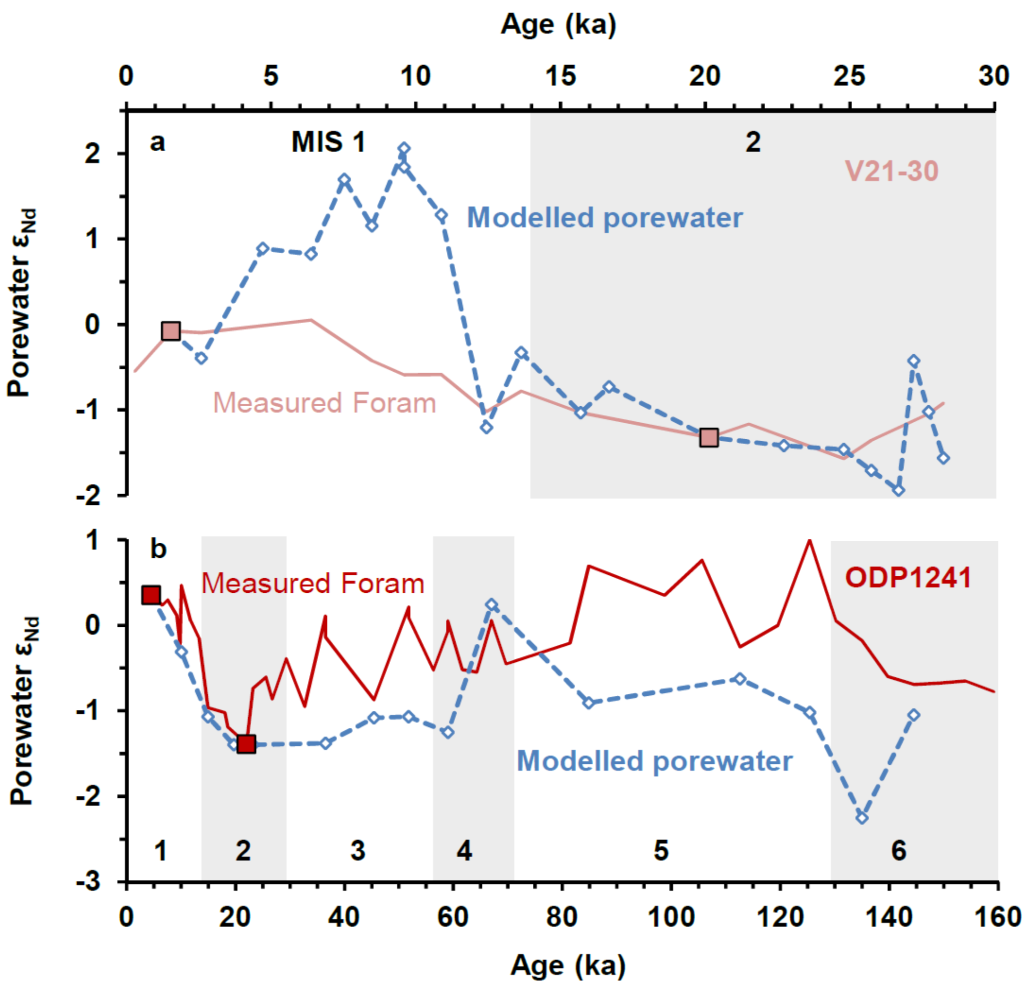

Supplementary Figure 3: Modelled porewater ε_{Nd} records (dashed curves) of V21-30 (a) and ODP1241 (b) in the sensitivity tests compared to foraminiferal ε_{Nd} records (solid curves). The solid squares mark the core-top and LGM datapoints whose $\varepsilon_{Nd\text{-}pw}$ values are made to match measured foraminiferal ε_{Nd} in equation (1) by tuning F_{au}, α_{vol} and α_{cont}. Similar relationships among the three unknowns are obtained for both cores: α_{vol} = ~0.5-0.6 α_{cont}, and F_{au} = ~8-9 × 10^{-5} × α_{cont}. Neither of these phenomena are realistic, because (1) the reactivity of volcanic material has shown to be greater than continental material; (2) assuming α_{cont} = 1-30%[ref1-4], the calculated F_{au} is lower than F_{mean} (even as low as one or two orders of magnitude). Despite the unrealistic parameters, the resultant modelled $\varepsilon_{Nd\text{-}pw}$ records are still not consistent with the foraminiferal data. Grey bars show the glacial MIS stages.

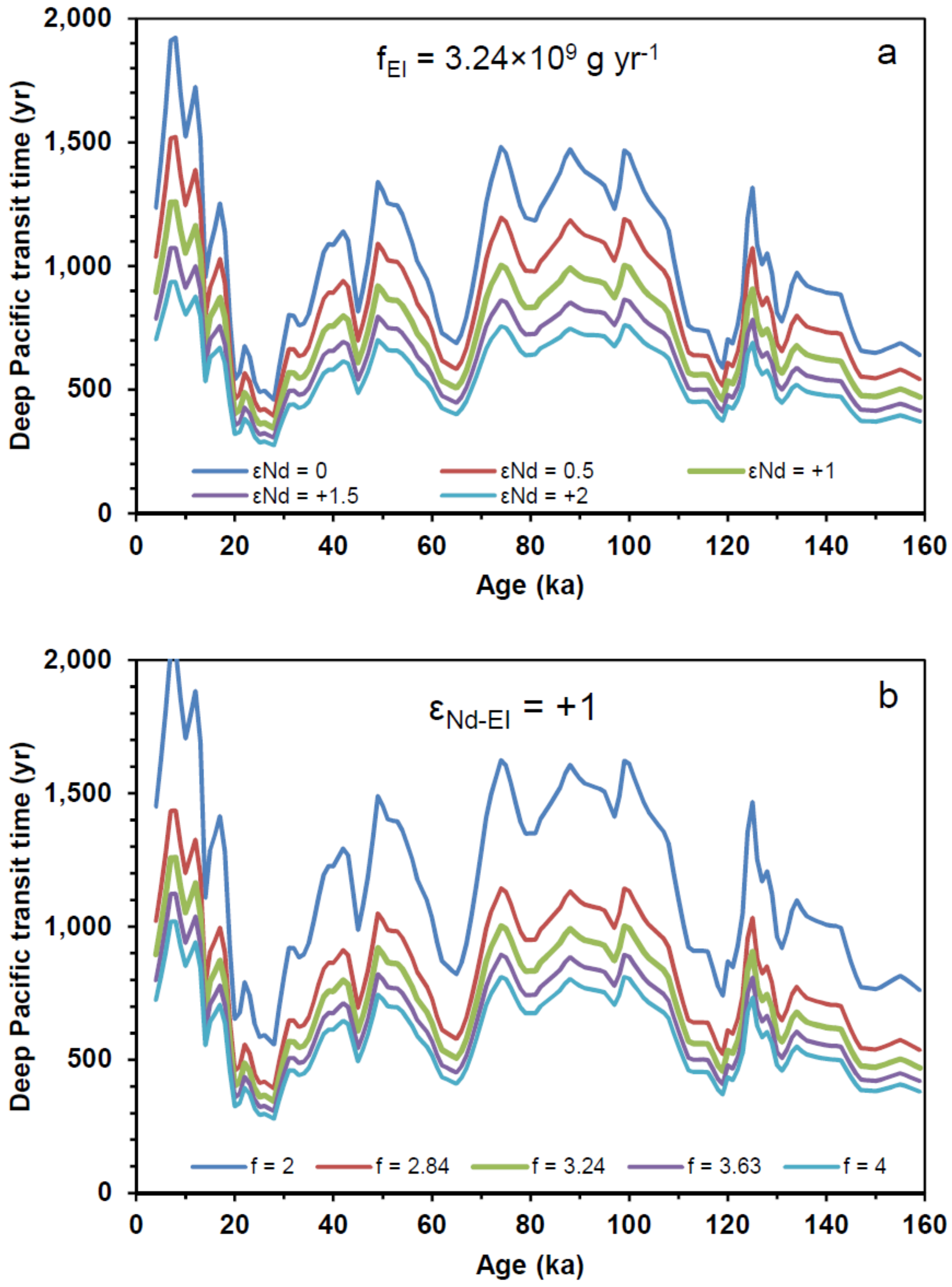

Supplementary Figure 4: Sensitivity tests of deep Pacific transit time. (a) Set the Nd flux of external input as $f_{EI} = 3.24\times10^9$ g yr^{-1}, and vary ε_{Nd} of external input: $\varepsilon_{Nd\text{-}EI}$ = 0, +0.5, +1, +1.5, +2. (b) Set the ε_{Nd} of external input as $\varepsilon_{Nd\text{-}EI}$ = +1, and vary the external input flux of Nd: f_{EI} = 2, 2.84, 3.24, 3.63, 4×10^9 g yr^{-1}. A clear pattern of shorter transit time approaching peak glaciation is shown in both cases.

Supplementary Tables

Supplementary Table 1: Locations of the cores used in this study.

No	Core location					Foraminiferal ε_{Nd}		
	Core	Latitude	Longitude	Water depth (m)	Geographic setting	Core-top ε_{Nd}[a]	2σ	Ref
	Eastern Equatorial Pacific							
1	V21-30	1°13’S	89°41’W	617	Galápagos Islands	-0.5	0.3	this study
2	ODP1241	5°51’N	86°27’W	2027	Cocos Ridge	0.4	0.2	this study
3	ODP846	3°5.8’S	90°49.07’W	3296	Peru Basin	-2.6	0.7	this study
4	RC13-114	1°39’S	103°38’W	3436	W. East Pacific Rise	-2.5	0.2	this study
	Western Equatorial Pacific							
5	MD01-2385	0.22°S	134.24°E	2602	Philippine Sea	-2.7	0.3	5
6	V28-239	3°15’N	159°11’E	3490	Solomon Plateau	-3.3	0.1	this study
	Southwest Pacific							
7	Y9	48° 14.21'S	177° 20.67'E	1267	Bounty Plateau	-7.0	0.2	6
8	SO136-38	50° 13.43'S	175° 18.73'E	1359	Bounty Plateau	-7.0	0.2	this study
9	CHAT16K	42º 32.00'S	178º 29.92'W	1408	N. Chatham Rise	-3.2	0.5	6, this study
10	CHAT10K	40º 01.97'S	179º 59.79'E	3003	N. Chatham Drift	-3.3	0.7	7
11	ODP1123[b]	41° 47.15'S	171° 29.94'W	3290	N. Chatham Drift	-6.8	0.3	8
12	CHAT1K	41°35'S	171° 30'W	3556	N. Chatham Drift			6
13	CHAT5K	40°47'S	171° 32.94'W	4240	N. Chatham Drift	-6.8	0.5	6, 7
14	CHAT3K	42º 39.59'	167º 29.77'W	4802	E. Chatham Drift			7
	Central South Pacific							
15	E11-2	56°S	115°W	3109	E. East Pacific Rise	-7.7	0.2	9
16	SO213-59-2	45°50’S	116°53’W	3161	W. East Pacific Rise	-6.1	0.2	10
17	SO213-60-1	44°58’S	119°33’W	3471	W. East Pacific Rise	-5.8	0.3	10

[a] None of the core materials have pristine core-tops, so the core-top foraminiferal ε_{Nd} are substituted by the ε_{Nd} values of the youngest Holocene materials of each sample.

[b] The Nd isotope record of ODP1123 combines existing data[8] with those of a nearby site CHAT1K[6].

Supplementary Table 2: Nd isotope record of V21-30.

Depth top (cm)	Depth bottom (cm)	Age (ka)	Foram ε_{Nd}	2σ	Detrital ε_{Nd}	2σ
0	5	0.3	-0.5	0.3		
13	14	1.5	-0.1	0.2	2.5	0.2
20	25	2.6	-0.1	0.2	2.0	0.1
40	45	4.7			3.6	0.2
60	65	6.4	0.1	0.2	3.3	0.2
80	85	7.5			4.0	0.2
80	85	7.5			3.9	0.2
100	105	8.5	-0.4	0.2	3.4	0.2
100	105	8.5			3.3	0.2
120	125	9.6	-0.6	0.2	4.4	0.2
120	125	9.6			4.2	0.2
140	145	10.9	-0.6	0.2	3.7	0.2
160	165	12.4	-1.0	0.2	1.0	0.2
180	185	13.6	-0.8	0.2	2.0	0.2
200	205	15.7	-1.0	0.2	1.2	0.2
220	225	16.7			1.5	0.2
240	245	20.1	-1.3	0.2	0.7	0.2
250	255	21.5	-1.2	0.2		
260	265	22.7			0.7	0.2
280	285	24.8	-1.6	0.2	0.6	0.2
280	285	24.8			0.5	0.2
300	305	25.7	-1.4	0.2	0.4	0.2
320	325	26.7			0.2	0.2
340	345	27.2			1.8	0.2
360	365	27.7	-1.0	0.2	1.2	0.2
380	385	28.2	-0.9	0.2	0.6	0.2

Supplementary Table 3: Nd isotope record of V28-239.

Depth top (cm)	Depth bottom (cm)	Age (ka)	Foram ε_{Nd}	2σ
4	5	2.5	-3.3	0.1
7	8	4.3	-3.2	0.1
10	11	6.2	-3.3	0.1
13	14	8.1	-3.2	0.1
15	16	9.3	-3.2	0.2
17	18	10.6	-3.0	0.2
20	21	12.4	-3.3	0.1
23	24	14.3	-3.5	0.1
25	26	15.5	-3.5	0.2
26	27	16.1	-3.3	0.1
28	29	17.4	-3.4	0.3
29	30	18.0	-3.5	0.2
30	31	18.9	-3.5	0.1
32	33	20.7	-3.6	0.1

35	36	23.5	-3.6	0.1
40	41	28.0	-3.5	0.1

Supplementary Table 4: Nd isotope record of SO136-38.

Depth top (cm)	Depth bottom (cm)	Age (ka)	Foram ε_{Nd}	2σ
3	4	2.5	-7.0	0.2
8	9	5.0	-6.7	0.2
13	14	7.5	-6.7	0.2
23	24	12.5	-6.3	0.2
28	29	14.8	-6.0	0.2
33	34	16.8	-5.8	0.2
38	39	18.9	-5.7	0.2
43	44	20.9	-5.9	0.2
48	49	22.9	-6.0	0.2
53	54	25.0	-6.3	0.2
58	59	27.0	-6.3	0.2
63	64	37.0	-6.1	0.2
68	69	47.0	-6.8	0.2
73	74	57.0	-7.0	0.2
78	79	60.9	-6.8	0.2
83	84	64.8	-6.8	0.2
88	89	68.7	-6.8	0.2
93	94	73.0	-7.4	0.2
98	99	78.0	-7.1	0.2
103	104	83.0	-7.0	0.2
108	109	88.0	-7.0	0.2
113	114	93.0	-6.8	0.2
118	119	98.0	-6.8	0.2
123	124	103.0	-7.0	0.2
128	129	108.0	-6.8	0.2
133	134	113.0	-6.8	0.2
138	139	118.0	-6.1	0.2
143	144	123.0	-6.2	0.2
148	149	128.0	-6.0	0.2
153	154	132.7	-6.1	0.2
158	159	137.1	-5.5	0.2
163	164	141.6	-5.5	0.2
168	169	146.0	-5.6	0.2
173	174	150.4	-5.7	0.2

Supplementary Table 5: Nd isotope record of CHAT16K.

Depth top (cm)	Depth bottom (cm)	Age (ka)	Foram ε_{Nd}	2σ

1	2	1.5	-3.2	0.5
5	6	3.6	-3.3	0.5
5	6	3.6	-3.6	0.3
9	10	5.7	-3.6	0.4
9	10	5.7	-3.9	0.2
16	17	9.3	-3.8	0.4
16	17	9.3	-4.0	0.2
25	26	14.0	-3.9	0.4
29	30	15.4	-4.0	0.4
33	34	16.8	-4.2	0.2
45	46	21.1	-3.4	0.2
48	49	22.2	-3.7	0.4
56	57	25.0	-3.6	0.5
61	62	26.6	-3.6	0.5
71	72	29.6	-4.1	0.2
81	82	32.7	-3.3	0.5
81	82	32.7	-4.1	0.2
81	82	32.7	-3.6	0.3
85	86	33.9	-4.0	0.2
89	90	35.1	-4.1	0.5
89	90	35.1	-3.8	0.2
97	98	37.5	-3.7	0.5
103	104	39.4	-3.7	0.5
111	112	41.8	-3.7	0.5
119	120	44.2	-3.5	0.5
119	120	44.2	-3.9	0.2
127	128	46.7	-3.5	0.4
131	132	47.9	-3.9	0.2
135	136	49.1	-3.5	0.4
139	140	50.3	-4.6	0.2
143	144	51.5	-3.7	0.5
151	152	54.0	-4.1	0.5
151	152	54.0	-3.5	0.5
159	160	56.4	-3.8	0.5
167	168	61.7	-3.1	0.4
175	176	67.9	-3.1	0.2
183	184	75.2	-3.9	0.6
191	192	83.6	-3.5	0.6
199	200	92.1	-3.0	0.2
207	208	100.5	-3.1	0.2
215	216	108.9	-3.1	0.2
223	224	117.4	-3.0	0.4
231	232	125.8	-3.4	0.4
239	240	131.9	-3.3	0.4
247	248	135.7	-3.7	0.6

255	256	139.5	-3.6	0.6
263	264	143.3	-3.9	0.6
263	264	143.3	-3.4	0.6
271	272	147.1	-3.5	0.6
279	280	151.1	-3.1	0.2
287	288	155.4	-3.6	0.2
295	296	159.7	-3.8	0.6
303	304	164.1	-3.6	0.6

Supplementary Table 6: Nd isotope record of ODP1241_Hole B_Core 1_Type H.

Site	Section	Top (cm)	Bottom (cm)	Age (ka)	Foram ε_{Nd}	2σ	Detrital ε_{Nd}	2σ
ODP1241B	1	10	12	4.5	0.4	0.2	4.4	0.2
ODP1241B	1	14	16	6.5	0.2	0.6		
ODP1241B	1	16	18	7.6	0.3	0.1		
ODP1241B	1	22	24	9.2	0.1	0.2		
ODP1241B	1	24	26	9.8	-0.2	0.4		
ODP1241B	1	25	27	10.1	0.5	0.2	2.6	0.1
ODP1241B	1	31	33	11.8	0.1	0.2		
ODP1241B	1	34	36	13.3	-0.2	0.4		
ODP1241B	1	37	39	14.9	-1.0	0.4	1.0	0.1
ODP1241B	1	43	45	18.1	-1.0	0.2		
ODP1241B	1	44	46	18.6	-1.2	0.2		
ODP1241B	1	46	48	19.7			0.0	0.2
ODP1241B	1	52	54	22.0	-1.4	0.3	0.5	0.2
ODP1241B	1	55	57	23.2	-0.7	0.2	0.4	0.1
ODP1241B	1	61	63	25.6	-0.6	0.2		
ODP1241B	1	64	66	26.8	-0.9	0.6		
ODP1241B	1	70	73	29.3	-0.4	0.2		
ODP1241B	1	76	78	32.7	-1.0	0.4		
ODP1241B	1	82	84	36.5	0.1	0.2	-0.1	0.2
ODP1241B	1	82	84	36.5	-0.1	0.4		
ODP1241B	1	96	98	45.4	-0.9	0.4	0.9	0.1
ODP1241B	1	106	108	51.8	0.2	0.2	0.9	0.1
ODP1241B	1	106	108	51.8	0.1	0.2		
ODP1241B	1	116	118	56.3	-0.5	0.3		
ODP1241B	1	126	128	59.0	-0.1	0.2	0.9	0.1
ODP1241B	1	126	128	59.0	0.1	0.2		
ODP1241B	1	136	138	61.7	-0.5	0.4		
ODP1241B	1	146	148	64.3	-0.5	0.2		
ODP1241B	2	6	8	67.0	0.0	0.2	3.4	0.1
ODP1241B	2	6	8	67.0	0.1	0.3	3.2	0.1
ODP1241B	2	16	18	69.7	-0.5	0.2		
ODP1241B	2	36	38	81.4	-0.2	0.3		
ODP1241B	2	41	43	84.9	0.7	0.6	1.4	0.1
ODP1241B	2	61	63	98.7	0.4	0.2		
ODP1241B	2	71	73	105.7	0.8	0.4		

ODP1241B	2	81	83	112.6	-0.3	0.1	2.2	0.2
ODP1241B	2	91	93	119.5	0.0	0.1		
ODP1241B	2	101	103	125.4	1.0	0.4	1.1	0.2
ODP1241B	2	101	103	125.4			1.2	0.2
ODP1241B	2	111	113	130.2	0.1	0.1		
ODP1241B	2	121	123	135.0	-0.2	0.1	-2.1	0.2
ODP1241B	2	121	123	135.0			-2.1	0.2
ODP1241B	2	131	133	139.8	-0.6	0.2		
ODP1241B	2	141	143	144.5	-0.7	0.1	1.1	0.2
ODP1241B	3	1	3	149.3	-0.7	0.1		
ODP1241B	3	11	13	154.0	-0.6	0.2		
ODP1241B	3	21	23	159.2	-0.8	0.2		

Supplementary Table 7: Nd isotope record of ODP846_Hole B/D_Core 1_Type H.

Site	Section	Top (cm)	Bottom (cm)	Age (ka)	Foram ε_{Nd}	2σ
ODP846B	1	9	14	4.0	-2.6	0.7
ODP846B	1	21	26	7.5	-1.8	0.7
ODP846B	1	31	35	9.9	-2.2	0.2
ODP846B	1	40	45	12.4	-2.0	0.2
ODP846B	1	50	55	15.0	-2.8	0.7
ODP846B	1	60	65	17.6	-2.1	0.6
ODP846B	1	70	75	19.9	-2.9	0.6
ODP846B	1	85	90	22.4	-2.5	0.7
ODP846B	1	85	90	24.3	-2.8	0.4
ODP846B	1	97	102	24.3	-2.7	0.3
ODP846B	1	110	115	26.3	-2.8	0.4
ODP846B	1	121	126	28.0	-3.0	0.3
ODP846B	1	130	135	29.4	-2.6	0.6
ODP846B	1	142	147	31.2	-2.4	0.4
ODP846B	2	6	11	33.4	-2.6	0.3
ODP846B	2	16	21	35.0	-2.5	0.3
ODP846B	2	35	38	38.5	-2.3	0.4
ODP846B	2	54	59	42.7	-2.4	0.4
ODP846B	2	65	70	45.0	-2.8	0.3
ODP846B	2	65	70	45.0	-3.1	0.2
ODP846B	2	85	90	49.1	-2.2	0.4
ODP846B	2	94	99	51.0	-2.2	0.4
ODP846B	2	95	100	51.2	-2.2	0.2
ODP846B	2	106	111	53.4	-2.2	0.7
ODP846B	2	125	130	57.0	-2.5	0.3
ODP846B	2	143	145	59.6	-2.5	0.3
ODP846B	3	6	11	61.8	-2.9	0.2
ODP846B	3	6	11	61.8	-2.7	0.2
ODP846B	3	25	30	64.8	-2.8	0.2

ODP846B	3	36	41	66.5	-3.0	0.3
ODP846B	3	36	41	66.5	-2.6	0.2
ODP846B	3	64	69	70.9	-2.3	0.3
ODP846B	3	76	80	74.1	-2.1	0.2
ODP846B	3	95	100	81.3	-2.4	0.2
ODP846B	3	115	120	87.7	-1.9	0.2
ODP846D	1	115	120	87.7	-2.2	0.2
ODP846D	1	125	130	97.5	-2.5	0.2
ODP846B	3	143	148	98.7	-2.1	0.3
ODP846D	1	145	150	107.8	-2.4	0.2
ODP846D	2	5	9	112.2	-3.0	0.2
ODP846D	2	12	16	115.9	-3.2	0.2
ODP846D	2	20	25	118.8	-3.5	0.2
ODP846D	2	25	30	120.2	-3.1	0.3
ODP846D	2	30	35	121.2	-3.2	0.2
ODP846D	2	40	45	123.3	-2.8	0.2
ODP846D	2	45	50	124.3	-2.2	0.4
ODP846D	2	50	55	125.3	-2.2	0.2
ODP846D	2	55	60	126.4	-2.6	0.2
ODP846D	2	65	70	128.4	-2.2	0.4
ODP846D	2	65	70	128.4	-2.5	0.2
ODP846D	2	75	77	130.2	-2.7	0.4
ODP846D	2	75	80	131.2	-2.8	0.2
ODP846D	2	75	80	131.2	-2.7	0.2
ODP846D	2	90	95	133.6	-2.3	0.2
ODP846D	2	110	115	137.8	-2.3	0.2
ODP846D	2	130	135	142.9	-2.3	0.2
ODP846D	2	145	150	146.9	-2.6	0.3
ODP846D	3	45	50	159.6	-2.8	0.3

Supplementary Table 8: Nd isotope record of RC13-114.

Depth top (cm)	Depth bottom (cm)	Age (ka)	Foram ε_{Nd}	2σ
0	2	3.2	-2.5	0.2
24	25	7.0	-1.9	0.4
41	42	12.2	-2.8	0.1
56	58	20.0	-2.5	0.4
161	162	52.6	-3.0	0.2
201	202	70.6	-2.3	0.4
296	298	119.9	-2.8	0.2
344	345	142.6	-3.0	0.1

Supplementary Table 9: Radiocarbon ages used to refine the age model of the upper part of ODP1241_Hole B_Core 1_Type H_Section 1.

Sample	Top (cm)	Bottom (cm)	Raw C-14 Age (yr)	Age error (± yr)	Calibrated age (yr)	Lab Accession Number
ODP1241B1H1	16	18	8,991	42	7,553	AA106608
ODP1241B1H1	31	33	12,381	49	11,752	AA106609
ODP1241B1H1	46	48	18,387	79	19,672	AA106610
ODP1241B1H1	55	57	18,641	82	19,982	AA106611

The AMS ^{14}C dates were measured at NSF-AMS Facility of Arizona University, USA. The radiocarbon dates were corrected for reservoir age of 540 years[11] and converted to calendar age using Marine13 Calibration curve[12] (http://calib.qub.ac.uk).

Supplementary Table 10: List of parameters, corresponding abbreviations, and their values used in this study.

No	Variable	Symbol	Value	Unit	Ref
Model for examination of detrital influence on porewater ε_{Nd} records					
1	Area of the Pacific	A	1.65E+08	km^2	13
2	Volume of the Pacific	V	7.E+08	km^3	13
3	Deepwater density	ρ	1023.6	$kg\ m^{-3}$	
4	Molecular mass of Nd	M_{Nd}	144.24	$g\ mol^{-1}$	
5	Nd residence time	τ	500	yr	14
6	Potential porewater Nd isotopic composition of sediment	$\varepsilon_{Nd\text{-}pw}$			
7	Authigenic Nd isotopic composition scavenged from seawater	$\varepsilon_{Nd\text{-}au}$			
8	Authigenic Nd isotopic composition for V21-30 equals to the ambient seawater ε_{Nd}	$\varepsilon_{Nd\text{-}au\text{-}2130}$	-1.1		15
9	Authigenic Nd isotopic composition for ODP1241 equals to the ambient seawater ε_{Nd}	$\varepsilon_{Nd\text{-}au\text{-}1241}$	-0.8		16
10	Nd isotopic composition of volcanic arc	$\varepsilon_{Nd\text{-}vol}$	+7		17
11	Nd isotopic composition of continental dust	$\varepsilon_{Nd\text{-}cont}$	-10		18
12	Detrital Nd isotopic composition of sediment	$\varepsilon_{Nd\text{-}d}$			
13	Authigenic particulate Nd flux	F_{au}		$g\ Nd\ m^{-2}\ yr^{-1}$	
14	Lithogenic flux of Nd	F_{litho}		$g\ Nd\ m^{-2}\ yr^{-1}$	
15	Volcanic flux of detrital Nd	F_{vol}		$g\ Nd\ m^{-2}\ yr^{-1}$	
16	Continental dust flux of detrital Nd	F_{cont}		$g\ Nd\ m^{-2}\ yr^{-1}$	
17	Mean authigenic Nd flux of the Pacific Ocean	F_{mean}	2.76E-05	$g\ Nd\ m^{-2}\ yr^{-1}$	this study
18	Dissolution factor of volcanic arc material	α_{vol}			
19	Dissolution factor of continental dust	α_{cont}	2%		1
20	Mean dissolved Nd concentration in the Pacific	C_d	22	$pmol\ kg^{-1}$	14
21	Detrital Nd concentration of EEP sediment	C_p	15	ppm	this study
22	Dry bulk density of V21-30 and ODP1241	DBD	0.63	$g\ cm^{-3}$	19
23	Linear sedimentation rate	LSR		$m\ yr^{-1}$	
24	Carbonate content in weight	X_{CaCO3}			
25	Proportions of continental particles in detrital component of the sediment	X_{cont}			
Model for estimation of deep Pacific transit time					
26	Amount of Nd in the water parcel	Q_{Nd}			
27	Amount of Nd brought by the LCDW inflow in the water parcel	Q_{LCDW}			
28	Nd concentration of LCDW	C_{LCDW}	24	$pmol\ kg^{-1}$	20
29	Reference Nd flux of external input to the Pacific Ocean	f_{EI}	3.24E+09	$g\ Nd\ yr^{-1}$	14, 21
30	Deep water transit time in the Pacific	t			
31	Nd isotopic composition of LCDW in the SWP (represented by ODP1123)	$\varepsilon_{Nd\text{-}LCDW}$			
32	Nd isotopic composition of PDW return flow in the EEP (represented by ODP846)	$\varepsilon_{Nd\text{-}RF}$			
33	Reference Nd isotopic composition of external input to the Pacific Ocean	$\varepsilon_{Nd\text{-}EI}$	+1		14

Supplementary References

1. Greaves, M.J., Statham, P.J. & Elderfield, H. Rare earth element mobilization from marine atmospheric dust into seawater. *Mar Chem* **46**, 255-260 (1994).
2. Henry, F., Jeandel, C., Dupré, B. & Minster, J.F. Particulate and dissolved Nd in the western Mediterranean Sea: Sources, fate and budget. *Mar Chem* **45**, 283-305 (1994).
3. Jeandel, C., Bishop, J.K. & Zindler, A. Exchange of neodymium and its isotopes between seawater and small and large particles in the Sargasso Sea. *Geochim Cosmochim Ac* **59**, 535-547 (1995).
4. Tachikawa, K., Jeandel, C., Vangriesheim, A. & Dupré, B. Distribution of rare earth elements and neodymium isotopes in suspended particles of the tropical Atlantic Ocean (EUMELI site). *Deep Sea Research Part I: Oceanographic Research Papers* **46**, 733-755 (1999).
5. Wu, Q. *et al.* Foraminiferal εNd in the deep north-western subtropical Pacific Ocean: Tracing changes in weathering input over the last 30,000 years. *Chem Geol* **470**, 55-66 (2017).
6. Hu, R. *et al.* Neodymium isotopic evidence for linked changes in Southeast Atlantic and Southwest Pacific circulation over the last 200 kyr. *Earth Planet Sc Lett* **455**, 106-114 (2016).
7. Noble, T.L., Piotrowski, A.M. & McCave, I.N. Neodymium isotopic composition of intermediate and deep waters in the glacial southwest Pacific. *Earth Planet Sc Lett* **384**, 27-36 (2013).
8. Elderfield, H. *et al.* Evolution of Ocean Temperature and Ice Volume Through the Mid-Pleistocene Climate Transition. *Science* **337**, 704-709 (2012).
9. Basak, C. *et al.* Breakup of last glacial deep stratification in the South Pacific. *Science* **359**, 900-904 (2018).
10. Molina-Kescher, M. *et al.* Reduced admixture of North Atlantic Deep Water to the deep central South Pacific during the last two glacial periods. *Paleoceanography* **31**, doi:10.1002/2015PA002863 (2016).
11. Fairbanks, R.G. *et al.* Radiocarbon calibration curve spanning 0 to 50,000 years BP based on paired ^{230}Th/ ^{234}U/ ^{238}U and ^{14}C dates on pristine corals. *Quaternary Science Reviews* **24**, 1781-1796 (2005).
12. Reimer, P.J. *et al.* IntCal13 and Marine13 radiocarbon age calibration curves 0–50,000 years cal BP. *Radiocarbon* **55**, 1869-1887 (2013).
13. World Atlas (2018). https://www.worldatlas.com.
14. Tachikawa, K., Athias, V. & Jeandel, C. Neodymium budget in the modern ocean and paleo-oceanographic implications. *J. Geophys. Res.* **108**, 3254 (2003).
15. Grasse, P., Stichel, T., Stumpf, R., Stramma, L. & Frank, M. The distribution of neodymium isotopes and concentrations in the Eastern Equatorial Pacific: Water mass advection versus particle exchange. *Earth Planet Sc Lett* **353–354**, 198-207 (2012).
16. Grasse, P. *et al.* Short-term variability of dissolved rare earth elements and neodymium isotopes in the entire water column of the Panama Basin. *Earth Planet Sc Lett* **475**, 242-253 (2017).

17. Grenier, M. *et al.* From the subtropics to the central equatorial Pacific Ocean: Neodymium isotopic composition and rare earth element concentration variations. *Journal of Geophysical Research: Oceans* **118**, 592-618 (2013).
18. Nakai, S.i., Halliday, A.N. & Rea, D.K. Provenance of dust in the Pacific Ocean. *Earth Planet Sc Lett* **119**, 143-157 (1993).
19. Mix, A.C., Tiedemann, R., Baldauf, J. & Blum, P. Site 1241. Proceedings of the Ocean Drilling Program, Initial Report **202**, IR-12 (2003).
20. Tachikawa, K. *et al.* The large-scale evolution of neodymium isotopic composition in the global modern and Holocene ocean revealed from seawater and archive data. *Chem Geol* **457**, 131-148 (2017).
21. Arsouze, T., Dutay, J.C., Lacan, F. & Jeandel, C. Reconstructing the Nd oceanic cycle using a coupled dynamical – biogeochemical model. *Biogeosciences* **6**, 2829-2846 (2009).
